# Chalcone Synthase-Encoding *AeCHS* is Involved in Normal Petal Coloration in *Actinidia eriantha*

**DOI:** 10.3390/genes10120949

**Published:** 2019-11-20

**Authors:** Yukuo Li, Wen Cui, Xiujuan Qi, Chengkui Qiao, Miaomiao Lin, Yunpeng Zhong, Chungen Hu, Jinbao Fang

**Affiliations:** 1 Zhengzhou Fruit Research Institute, Chinese Academy of Agricultural Sciences, Zhengzhou 450009, China; liyukuokiwi@126.com (Y.L.);; 2 Key Laboratory of Horticultural Plant Biology, College of Horticulture & Forestry Sciences of Huazhong Agricultural University, Wuhan 430000, China

**Keywords:** anthocyanin, infiltration grafting, structural gene, petal coloration, *Actinidia eriantha*

## Abstract

Studies on anthocyanin biosynthesis have been mainly concentrated on the fruit, whereas few have focused the mechanism of flower coloration in kiwifruit. Here, we report that the structural gene, *AeCHS*, is involved in anthocyanin accumulation and indispensable for normal petal coloration in *Actinidia eriantha*. Petals from three different species including *Actinidia eriantha* (red petals), *Actinidia hemsleyana* (light pink petals) and *Actinidia arguta* (white petals) were selected for anthocyanin determination and gene expression analysis. The anthocyanin components in *A. eriantha* were significantly higher than in *A. hemsleyana* or *A. arguta*. Consistently, gene expression profiles suggested that *AeCHS* expression in *A. eriantha* was higher than in *A. hemsleyana* or *A. arguta*. Cluster analysis showed that *AeCHS* was clustered into a single group and distinctly separated from other genes, indicating the expression pattern of *AeCHS* gene was different from any other. Additionally, correlation analysis revealed *AeCHS* expression significantly correlated with anthocyanin content. The complete coding sequence of *AeCHS* was cloned from petals of *A. eriantha* ‘Zaoxu’, showing the length of *AeCHS* was 1170 bp encoding a protein of 389 amino acids. *AeCHS* was located in the cytoplasm, indicating it is indeed a structural gene involved in anthocyanin biosynthesis. *AeCHS* silencing performed by infiltration grafting-mediated virus-induced gene silencing (VIGS) reduced petal anthocyanin content and bleached red petals in *A. eriantha*. Our results confirm a crucial role of *AeCHS* in anthocyanin biosynthesis and accumulation in *A. eriantha* petals; furthermore, they offer important basic information and constitute a reference point for further research.

## 1. Introduction

Kiwifruit belongs to the genus *Actinidia* of the Actinidiaceae. It is a perennial deciduous fruit tree originated in China [1] that comprises over 70 species that vary extensively with regard to fruit type and flowers, most of them distributed in China [2]. The commercial cultivation of kiwifruit, one of the four fruit trees successfully domesticated in the last century, has extended around the world [3]. The most important tissue in most fruit trees is ripened fruit flesh, because of their unique flavor and delicious rich taste, which is highly valued in the market and therefore, of significant economic benefit for producers. Thus, plant breeders are committed to developing new cultivars to cater for the market demand. ‘Hayward,’ the most popular kiwifruit cultivar, has dominated global markets for many years [4]. 

In addition to the fruit, flowers are important during the growth and development of the kiwifruit tree. Several *Actinida* species, such as *A. eriantha*, *A. hemsleyana*, *A. longicarpa*, *A. arguta*, *A. polygama*, among others, show red, pink, or white petals at full bloom [5]. *A. eriantha* with bright colored petals has the potential to be a suitable ornamental kiwifruit tree whose promotion and development has recently started. 

As is the case with red fruit coloration [6,7], red kiwifruit flowers are also due to the biosynthesis and accumulation of anthocyanins [8,9], a kind of water-soluble pigments produced by a branch in the flavonoid pathway that seemingly play an important role as attractants of insect and animal pollinators and seed dispersers [10,11]. Further, as natural food pigments, anthocyanins are of importance not only as plant endogenous compounds involved in a variety of biological responses but as natural plant extracts with enormous potential for human health as well [12,13,14,15,16,17]. The Anthocyanin biosynthetic pathway and the related genes including structural and regulatory genes have been extensively studied in plants [18,19,20,21,22,23,24,25,26,27,28,29,30].

**Figure 1:**
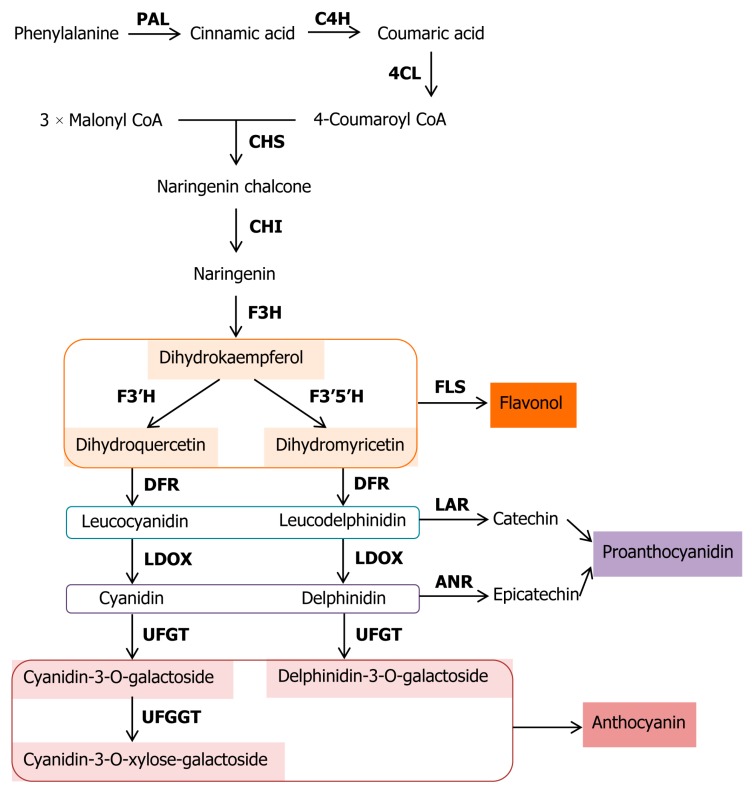
Anthocyanin biosynthetic pathway in kiwifruit. PAL, phenylalanine ammonia-lyase; C4H, trans-cinnamate 4-hydroxylase; 4CL, 4-coumarate: CoA ligase; CHS, chalcone synthase; CHI, chalcone isomerase; F3H, flavanone 3-hydroxylase; F3′H, flavonoid 3′-hydroxylase; F3′5′H, flavonoid 3′,5′-hydroxylase; DFR, dihydroflavonol 4-reductase; LDOX, leucoanthocyanidin dioxygenase; UFGT, flavonoid 3-O-galactosyl transferase; UFGGT, flavonoid 3-O-galactoside-xylosyl transferase; FLS, flavonol synthase; LAR, leucoanthocyanidin reductase; ANR, anthocyanidin reductase.

We have summarized schematically the components of the pathway possibly occurring in kiwifruit in Figure 1, based on previous research on the biosynthetic pathway of anthocyanin [6,31,32]. Similarly, previous studies showed that transcript abundance of most structural genes related to anthocyanin synthesis, such as *CHS* (encoding chalcone synthase), *F3H* (encoding flavanone 3-hydroxylase), *DFR* (encoding dihydroflavonol-4-reductase), and *UFGT* (encoding UDP-glucose: flavonoid 3-O-glycosyltransferase), are highly correlated with anthocyanin accumulation [33,34,35]. 

Numerous studies have focused on anthocyanin biosynthesis in fruits, but few on the mechanism of flower coloration. The overexpression of *SVP3* gene in kiwifruit affects reproductive development and suppresses anthocyanin biosynthesis in petals [9]. In addition, an R2R3 MYB transcription factor (MYB110a) reportedly determined red petal color in a kiwifruit hybrid population [8]. These studies provide a molecular basis for anthocyanin regulation of red petal coloration in kiwifruit; however, the specific structural gene that plays a decisive role in the formation of red petals remains elusive.

With the advent of the post-genomic and molecular biological era, an increasing number of combinations of omics and experimental systems are applied to unravel various biological phenomena. In kiwifruit, two *Actinidia* species including *Actinidia chinensis* and *Actinidia eriantha* have allowed the completion of genome sequencing [36,37,38], thus providing a sequence basis for the study of the molecular mechanism underlying various biological traits. Nonetheless, functional genomic studies in kiwifruit are still seriously limited compared with other model plants because of the late start of molecular biology techniques and the long period of the genetic transformation system in this case, where virus-induced gene silencing (VIGS) may be an ideal method to perform verification experiments of gene function by silencing the correct target gene. VIGS used for functional characterization of genes has been extensively reported in various plant species, including tobacco [39,40], rose [41,42,43], cotton [44], pea [45], potato [46], tomato [47,48,49], apple [50], and strawberry [51]. Furthermore, VIGS was recently successfully used in kiwifruit [52]. 

As perennial woody plants, grafting is the main reproductive mode for fruit trees, with the survival rate of grafts depending on the affinity of rootstocks and scions [53]. Therefore, the combination of grafting technology and VIGS may be a useful strategy to obtain information about a gene function of interest. Therefore, we used infiltration grafting-mediated VIGS to verify the role of a target gene in kiwifruit petal coloration. 

To explore possible molecular mechanisms underlying petal coloration, a series of physiological and biochemical experiments were carried out as well as a new try of infiltration grafting-mediated VIGS, providing new insight for anthocyanin biosynthesis and accumulation of kiwifruit petals.

## 2. Materials and Methods

### 2.1. Flower Materials

**Figure 2:**
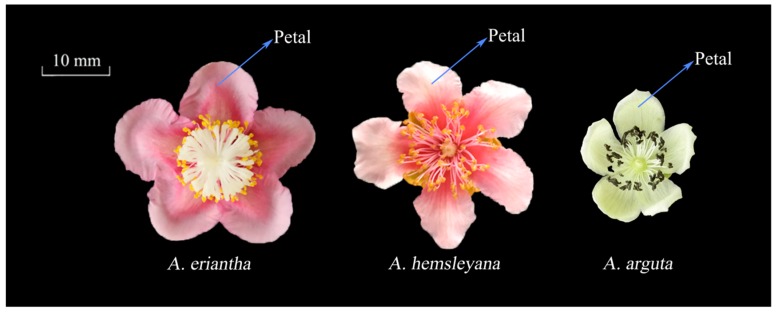
Flower phenotype in three different *Actinidia* species. The petal color of *A. eriantha*, *A. hemsleyana*, and *A. arguta* are red, light pink, and white, respectively.

Three types of flowers of different color, from three different *Actinidia* species including *Actinidia eriantha* ‘Zaoxu’, *Actinidia hemsleyana* NY-CY’, and *Actinidia arguta* ‘XX-RZ’, were collected from the National Kiwifruit Germplasm Garden (34.71569° N, 113.7122° E), at the Zhengzhou Fruit Research Institute of the Chinese Academy of Agricultural Sciences, Henan Province, China. The color of the petals of the three types of flowers at full blooming stage were red, light pink and white, respectively (Figure 2). For each *Actinidia* species, approximately 60 flowers were sampled from three independent vines. The petals were separated from the flowers, immediately frozen in liquid nitrogen, and stored at −80 °C until use for anthocyanin and RNA extraction.

For grafting experiments, branches with one unopened bud to be used for scions were cut from the adult *Actinidia eriantha* vine and terminal part was removed. *Actinidia valvata* twigs were used as rootstocks. Scions were grafted onto rootstocks by the traditional cleft grafting method and wrapped in transparent bags to keep high humidity conditions until bud opening.

### 2.2. Extraction and Determination of Anthocyanins and Precursors

Considering that anthocyanins are sensitive to light and easy to degrade, the whole extraction process was carried out under dark conditions. The extraction procedure was carried out according to the modified protocol for the determination of anthocyanins in products of plant origin, of the Agricultural Industry Standards of the People’s Republic of China (NY/T2640-2014). Briefly, approximately two grams of petal tissue were ground and extracted in an 80:20:1 solution t of anhydrous ethanol, water, and formic acid. The extracts were passed through a syringe filter with a 0.45-µm filter membrane (Jinteng, Tianjin, China) prior to chromatographic analysis. Qualitative and quantitative determination of anthocyanins and their precursors were conducted by ultra-performance liquid chromatography coupled with tandem mass spectrometry (UPLC-MS/MS) (Agilent Technologies Inc., CA, USA). Dihydroquercetin (CAS: 480-18-2), dihydromyricetin (CAS: 27200-12-0), cyanidin (CAS: 528-58-5), delphinidin (CAS: 528-53-0), cyanidin-3-O-galactoside (CAS: 27661-36-5), cyanidin-3-O-xylose-galactoside (CAS: 31073-32-2), and delphinidin-3-O-galactoside (CAS: 28500-00-7) were used as authentic standards for constructing the corresponding standard curves for single point quantitation. 

In petal samples used for VIGS, measurements of total anthocyanin content were carried out using the Plant Anthocyanin Content Detection Kit (Solarbio, Beijing, China) according to manufacturer instructions.

### 2.3. Total RNA Extraction, cDNA Synthesis, and qRT-PCR Analysis

Total RNA was extracted from petals using the Quick RNA Isolation Kit (Huayueyang, Beijing, China) in accordance with manufacturer instructions. The integrity and concentration of RNA were assessed and determined by 1% agarose gel electrophoresis and micro ultraviolet spectrophotometry using NanoDrop 2000 (Thermo Fisher Scientific, MA, USA), respectively. Approximately 1 µg of total RNA was used for cDNA synthesis using RevertAid™ First Strand cDNA Synthesis Kit (Thermo Fisher Scientific, MA, USA) according to manufacturer instructions. Fifteen genes encoding enzymes involved in anthocyanin biosynthesis were obtained from *Actinidia chinensis* cv ‘Red 5′ [37] or ‘Hongyang’ genomes [36]. Primers used for quantitative real-time polymerase chain reaction (qRT-PCR) were designed using Primer-Blast online tool in the National Center for Biotechnology Information (NCBI, https://www.ncbi.nlm.nih.gov/tools/primer-blast/). The specific primer sequences of these fifteen genes used for qRT-PCR are listed in Table 1. The 20-µL reaction mixture contained 5 µL of double distilled water, 10 µL of SYBR Green I Master Mix (Roche, Basel, Switzerland), 1 µL of forward and reverse primers for each gene, and 3 µL of cDNA template (diluted 40 times). The LightCycler^®^ 480 realtime PCR system (Roche, Basel, Switzerland) with a 96-well plate was used for qRT-PCR, and three biological replicates were included for each condition. Kiwifruit *β-actin* served as reference gene for normalization [54]. Relative quantification of gene expression level was performed using the 2(-Delta C(T)) (2^−ΔΔCt^) method as described previously [55].

### 2.4. Cloning and Sequencing of AeCHS

The coding sequence of *CHS* (DTZ79_22g06860) from the genome of *Actinidia eriantha* ‘White’ was selected as reference to design a primer [38]. The coding sequence (CDS) of *AeCHS* was amplified from *A. eriantha* ‘Zaoxu’ red petal sample by specific primers 5′-ATGGTGACTGTCGAGGAAGTTC-3′(forward) and 5′-CTAAGTGCACAGGCTATGGAGC-3′(reverse) using high-fidelity DNA polymerase KOD-Plus-Neo (TOYOBO, Osaka, Japan). The amplified product was inserted into the pMD18-T vector (TsingKe Biological Technology, Beijing, China) by using the TA cloning method. The construct was transformed to *E. coli* DH5α competent cells cultured on LB agar plates with ampicillin and incubated at 37 ℃. After PCR detection, positive clones were sequenced by the Sanger system (Sangon Biotech, Shanghai, China).

### 2.5. Subcellular Localization

The CDS of *AeCHS* without the stop codon was amplified from *A. arguta* by specific primers 5′-CCCAAGCTTGGGATGGTGACTGTCGAGGAAGTTC-3′ (forward primer) and 5′-TGCCTGCAGGCAAGTGCACAGGCTATGGAGC-3′ (reverse primer) containing *Hind* III and *Pst* I restriction enzymatic sites. The PCR product was recombined with the plant binary expression vector pHB to form CaMV 2×35S:AeCHS:YFP. The empty pHB vector only with the YFP gene (2×35S:YFP) was used as positive control. Two constructs were introduced into *A. tumefaciens* strain EHA105 using a freeze-thaw method. *A. tumefaciens* strains were kept at 28 ℃ in LB medium supplemented with kanamycin, resuspended in infiltration buffer containing 10 mM MgCl_2_, 10 mM MES and 200 µM acetosyringone to an OD_600_ of 0.6–1.0, and left to stand for 2 h at room temperature before infiltration. Subsequently, the *A. tumefaciens* solution was infiltrated into 5–6-week-old *N. benthamiana* leaves with a 1-ml needleless syringe. Infiltrated plants were placed under dark conditions at room temperature for the first 24 h and then under 16 h light and 8 h dark for another 24 h; YFP fluorescence was observed with a Leica TCS SP5 confocal laser scanning microscope (Leica Microsystems, Wetzlar, Germany).

### 2.6. Construction of Silencing Vector for Virus-induced Gene Silencing (VIGS)

Two types of TRV vector, pTRV1 and pTRV2, were selected for the VIGS experiment [47]. A 252 bp specific fragment of *AeCHS* coding sequence was amplified from *Actinidia eriantha* ‘Zaoxu’ cDNA by PCR using specific primer 5′-CCGGAATTCCGG CCTGCTATTTTGGACCAAGTGG-3′ (forward primer) and 5′-CGGGGTACCCCG CTAAGTGCACAGGCTATGGAGC-3′ (reverse primer) containing *Eco*R Ι and *Kpn* Ι restriction enzymatic sites (Figure S1). This fragment was cloned into the *Eco*R Ι and *Kpn* Ι sites of the pTRV2 vector to generate the pTRV2:AeCHS vector. The empty pTRV1 and pTRV2 vectors were used as controls. Three constructs were introduced into *A. tumefaciens* strain GV3101 using a freeze-thaw method, and then stored at −80 ℃ for further use.

### 2.7. Infiltration Grafting-mediated VIGS Experiment

For the virus-induced gene silencing (VIGS) experiment in *A. eriantha* petal, a modified infiltration grafting method was performed based on a previous study [43]. Briefly, *A. tumefaciens* strain GV3101 containing three TRV vectors including pTRV1, pTRV2 and pTRV2:AeCHS were grown at 28 ℃ for 16–24 h in LB medium (10 mM MES, 100 µM AS) supplemented with kanamycin and rifampicin. Activated *A. tumefaciens* cells were collected by centrifugation for 5 min at 1000 g, and resuspended in infiltration buffer (10 mM MgCl_2,_ 10 mM MES, 400 µM AS). Scions with a newly cut wound were submerged in 1:1 infiltration mixture consisting of pTRV1 and pTRV2:AeCHS (OD_600_, 0.8), and subjected to vacuum (DZF-60, RongFeng, Shanghai, China) at −25 kPa for 5 min. The 1:1 mixture of pTRV1 and pTRV2 was used as control. Treated scions were grafted onto *Actinidia valvata* twigs to use as rootstocks. 

### 2.8. Graph Making and Statistical Analysis

GraphPad Prism5 (GraphPad Software Inc., San Diego, CA, USA) was used for chart preparation. R-3.4.2 was used to obtain the heat map and to conduct cluster analyses. DNAman software (Lynnon Biosoft, USA) was used for sequence alignment. Data are means and standard deviations and were analyzed using Student’s *t*-tests. Differences among different groups were considered statistically significant at *P* ≤ 0.05. IBM SPSS Statistics 20 (IBM, New York, USA) was used to test significant differences.

## 3. Results

### 3.1. Phenotype Observation and Anthocyanin Content Analysis

**Figure 3:**
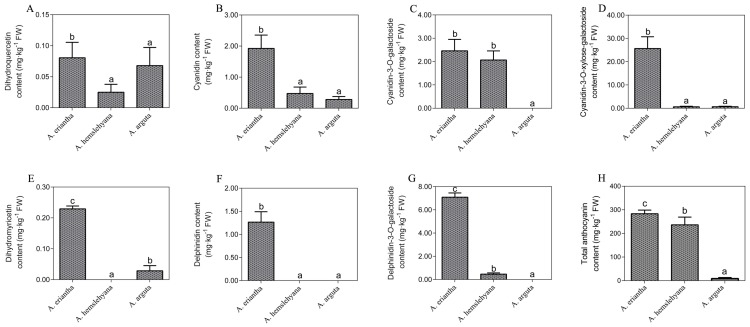
Anthocyanin components contents and their precursors including dihydroquercetin, dihydromyricetin, cyanidin, delphinidin, cyanidin-3-O-galactoside, delphinidin-3-O-galactoside and cyanidin-3-O-xylose-galactoside. (**A**) Dihydroquercetin content in three typed petals. (**B**) Cyanidin content in three typed petals. (**C**) Cyanidin-3-O-galactoside content in three typed petals. (**D**) Cyanidin-3-O-xylose-galactoside in three typed petals. (**E**) Dihydromyricetin content in three typed petals. (**F**) Delphinidin content in three typed petals. (**G**) Delphinidin-3-O-galactoside in three typed petals. (**H**) Total anthocyanin content in three typed petals. Data are means ± SE of three replicates. Error bars represent standard error of means. Columns with different lowercase letters are significantly different at *P* ≤ 0.05. Data were analyzed with Student’s *t*-test.

Phenotype observation and comparison among the three kinds of *Actinidia* petals revealed that *A. eriantha* petal color were of a more intense and darker red color than the other two *Actinidia* species *A. hemsleyana* and *A. arguta* (Figure 2). Analysis of anthocyanins and their precursors by UPLC-MS/MS revealed significant differences in the content of seven anthocyanin components among the three types of petals under study. Seven components were detected in *A. eriantha* petals whose content were significantly higher than those recorded for *A. hemsleyana* and *A. arguta.* In *A. eriantha* petals, cyanidin-3-O-xylose-galactoside content was the highest, reaching 25.65 mg kg^−1^ FW (Figure 3D), followed by delphinidin-3-O-galactoside (Figure 3G) and cyanidin-3-O-galactoside (Figure 3C). In *A. hemsleyana* and *A. arguta* petals, only four to five components were detected, and at very low levels (Figure 3A-G). This result correlated well with the visible phenotype, indicating anthocyanins, especially cyanidin-3-O-xylose-galactoside, seems to be associated with the formation of red color in petals. The total anthocyanin content in *A. eriantha* was significantly higher than that in *A. hemsleyana* and *A. arguta* (Figure 3H), which was consistent to petal phenotype.

### 3.2. Expression and Cluster Analysis of Genes Involved in Anthocyanin Biosynthesis

**Figure 4:**
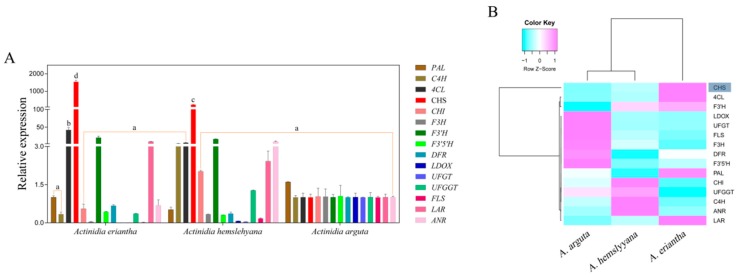
Expression profiles of structural genes in three *Actinidia* species including *A. eriantha*, *A. hemsleyana*, and *A. arguta* (**A**), and cluster analysis of gene expression (**B**). Data are means ± SE of three replicates. Error bars represent standard error of the means. Columns with different lowercase letters are significantly different at *P* ≤ 0.05. Cyan and pink boxes with normalized color scales from -1 to 1 indicate low and high expression level, respectively. The blue shadow indicates that *CHS* gene is clustered into a single group.

To investigate the cause of the difference in red, light pink, and white petals, petal samples of the three *Actinidia* species were used to analyze the gene expression level by qRT-PCR. The expression profile of anthocyanin biosynthetic genes, including *PAL*, *C4H*, *4CL*, *CHS*, *CHI*, *F3H*, *F3′H*, *F3′5′H*, *DFR*, *LDOX*, *UFGT*, *UFGGT*, *FLS*, *LAR*, and *ANR*, were investigated (Figure 4A). Except for two genes, namely, *4CL* and *CHS*, whose expression was very high in *A. eriantha*, most of the other genes showed no obvious expression rules. Only one gene, *CHS*, was expressed to significantly high levels in *A. eriantha*, thus reaching significant differences with respect to the other genes analyzed. In order to find out the expression patterns of genes involved in anthocyanin biosynthesis, cluster analysis was performed by the R-3.4.2 language package. Cluster analysis showed that *CHS* was clustered into a single class and was distinctly separated from the rest of the genes (Figure 4B), possibly indicating that the expression pattern of *CHS* was different from others. In addition, correlation analysis revealed that *AeCHS* expression was significantly correlated to anthocyanin content (Table 2), which clearly indicates *CHS* might be the key gene controlling anthocyanin biosynthesis in *A. eriantha* petals. 

### 3.3. Sequence Analysis and Alignment of AeCHS

**Figure 5:**
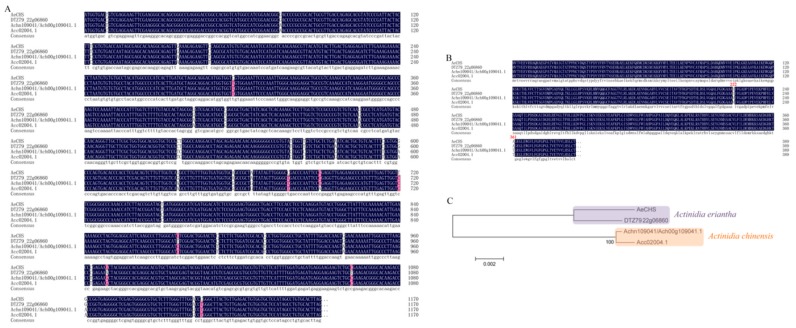
Sequence alignments of DNA and protein, and construction of phylogenetic tree. (**A**) DNA sequence alignment of *CHS* in different *Actinidia* species and cultivars. (**B**) Protein sequence alignment of CHS in different *Actinidia* species and cultivars. (**C**) Construction of the phylogenetic tree based on amino acid sequences. *AeCHS*, the complete coding sequence was identified and cloned from *Actinidia eriantha* ‘Zaoxu’ petals. DTZ79_22g06860, gene ID of *CHS* in *Actinidia eriantha* ‘White’; Achn109041/Ach00g109041.1, Gene ID of *CHS* in *Actinidia chinensis* ‘Hongyang’; Acc02004.1, Gene ID of *CHS* in *Actinidia chinensis* ‘Red 5′.

As mentioned above, *CHS* might be the key gene controlling anthocyanin biosynthesis in *A. eriantha* petals. Therefore, the full-length cDNA of *AeCHS* was cloned from *A. eriantha* ‘Zaoxu’ petals. The length of *AeCHS* is 1170 bp encoding a protein of 389 amino acids. A comparison of the cDNA sequences showed *CHS* had nucleotide divergence in *Actinidia* species or cultivars, including *A. eriantha* ‘Zaoxu,’ *A. eriantha* ‘White,’ *A. chinensis* ‘Hongyang’ and *A. chinensis* ‘Red 5′ (Figure 5A). Protein alignment suggested that there were two amino acid differences between *A. eriantha* and *A. chinensis* (Figure 5B), and phylogenesis showed that *A. eriantha* and *A. chinensis* were clustered into one class, (Figure 5C), which indicated CHS is different in different *Actinidia* species.

### 3.4. Subcellular Localization Analysis of AeCHS

**Figure 6:**
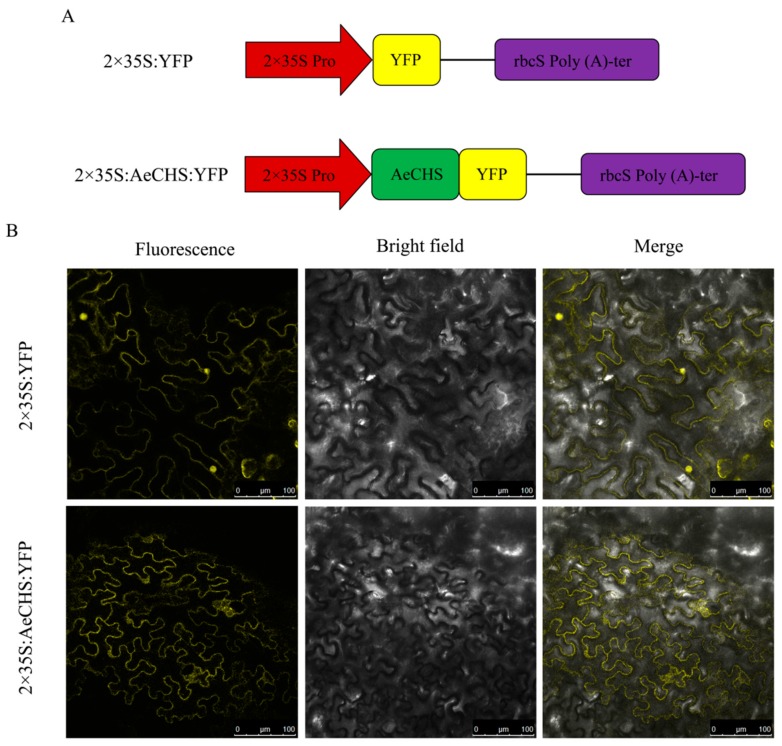
Subcellular localization. (**A**) Vector construction of 2×35S:YFP and 2×35S:AeCHS:YFP. (**B**) Subcellular localization of AeCHS in *Nicotiana benthamiana* leaves. Experiments were repeated three times. Cells expressing *AeCHS:YFP* fusion gene showed fluorescence in cytoplasm. Cells expressing empty plasmid with YFP tag was used as a control.

To determine the subcellular localization of the AeCHS protein, AeCHS was first predicted by online tool PSORT (https://www.genscript.com/tools/wolf-psort). After putting AeCHS protein sequence into retrieval system, corresponding prediction results that called neighbors were presented in online page. Prediction results showed that 12 neighbors were located in cytoplasm among 14 nearest neighbors, which indicated that the AeCHS protein was located in the cytoplasm. To verify this result, a subcellular localization experiment was performed by transient injection assays in *N. benthamiana* leaves. Vectors inserted with AeCHS:YFP and only YFP were constructed (Figure 6A). The positive control 2×35S:YFP was diffuse in the cytoplasm and concentrated in the nucleus. In contrast, the 2×35S:AeCHS:YFP fusion protein signal was detected only in the cytoplasm (Figure 6B), which indicated that *AeCHS* seemed likely to be a structural gene encoding chalcone synthase controlling anthocyanin biosynthesis. The same results were obtained in three independent experiments. 

### 3.5. Silencing of AeCHS in A. eriantha Petals

**Figure 7:**
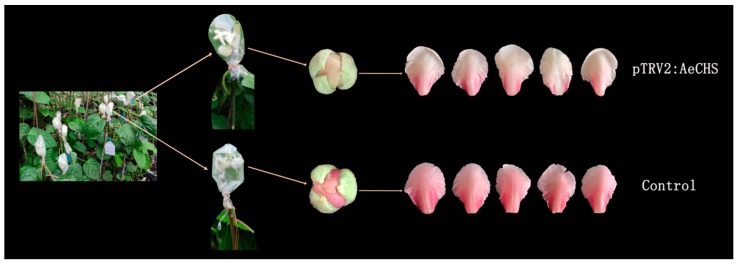
Petals of *AeCHS*-silenced (upper) and the control (lower) *A. eriantha* ‘Zaoxu’ flowers. Petals of *AeCHS*-silenced flowers were of a paler pink than those of the control, and close to white.

**Figure 8:**
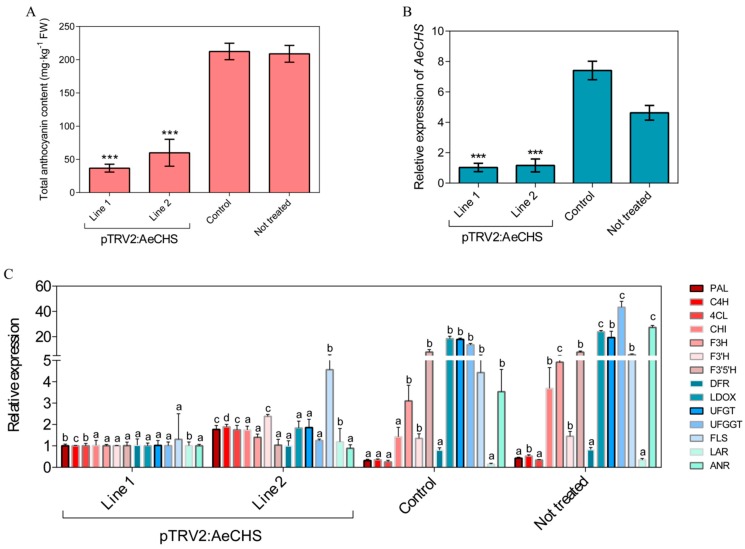
Total anthocyanin content and expression level of *AeCHS* and other genes involved in anthocyanin biosynthesis for VIGS samples. (**A**) Total anthocyanin analyses in silenced lines 1 and 2, control and untreated petal samples. (**B**) Expression profiles of *AeCHS* in silenced lines 1 and 2, control and untreated petal samples. Data are means ± SE of three replicates. Asterisks indicate significant differences calculated using Duncan’s test (*** *P* ≤ 0.001). (**C**) Expression level of related genes involved in anthocyanin biosynthesis in silenced lines 1 and 2, control and untreated petal samples. Different letters denote statistical significance using one-way ANOVA, *P* < 0.05.

Available approaches to gene silencing are limited and rarely reported in *Actinidia*. A novel approach, namely, an infiltration grafting-mediated VIGS system was applied in *A. eriantha* petals. The results showed *A. eriantha* ‘Zaoxu’ scions infiltrated with pTRV1/pTRV2:AeCHS virus displayed bleached petals at about twenty days after infiltration grafting. In contrast, control scions infiltrated with pTRV1/pTRV2 displayed normal petal color (Figure 7). In addition to phenotypic changes resulted from *AeCHS* silencing, pigment content and gene expression were analyzed to investigate whether silencing of *AeCHS* could induce other changes at molecular level in *A. eriantha* ‘Zaoxu’ petals. Total anthocyanin content in pTRV1/pTRV2:AeCHS infiltrated petals was significantly lower than that in pTRV1/pTRV2 infiltrated petals (Figure 8A). Transcript abundance of *AeCHS* was reduced in pTRV1/pTRV2:AeCHS infiltrated petals by approximately 85%, reaching a highly significant difference at 0.001 probability level (Figure 8B). Besides *AeCHS*, the relative expression level of other genes involved anthocyanin biosynthesis showed different changing trend in different typed petal samples. Compared to control and not treated petals, the expression of *LBGs* (late synthetic genes) including *LDOX*, *UFGT* and *UFGGT* was lower in silencing petals (Figure 8C).

## 4. Discussion

### 4.1. Anthocyanin Changes in Three Types of Petals

Anthocyanins dominate flower coloration in most plant species. We measured the concentrations of anthocyanins and their precursors in three types of petals including, *A. eriantha*, *A. hemsleyana*, and *A. arguta*, with red, light pink, and white color petals, respectively. The content of different anthocyanins components showed similar variation trends in the three types of petals. Except for cyanidin-3-O-galactoside, the content of other anthocyanins and their precursors in *A. eriantha* petals was significantly higher than that in *A. hemsleyana* or *A. arguta*. In addition, the concentration of cyanidin-3-O-xylose-galactoside was significantly higher than that of other anthocyanins and anthocyanins precursors, followed by delphinidin-3-O-galactoside and cyanidin-3-O-galactoside. These results not only indicate that the formation of the red color of petals is due to accumulation of anthocyanin components, additionally, they suggest that cyanidin-3-O-xylose-galactoside is the main anthocyanin component primarily responsible for the red color in petals of *A. eriantha*, which partially agrees with Fraser [8], who suggested that the red petal phenotype in the interspecific *Actinidia* population results from a mixture of anthocyanins, with cyanidin-3-O-galactoside present in the greatest concentration, and with cyanidin-3-O-xylose-galactoside and cyanidin-3-O-glucoside also present in significant amounts. However, the reasons for this difference need to be further explored.

### 4.2. Screening, Cloning and Subcellular Localization of AeCHS

Anthocyanin biosynthesis has been extensively studied in different plant tissues including fruits, flowers, and seeds, among others. Anthocyanin accumulation is determined by structural genes including, *PAL*, *C4H*, *4CL*, *CHS*, *CHI*, *F3H*, *F3′H*, *F3′5′H*, *DFR*, *LDOX*, *UFGT*, *UFGGT*, *FLS*, *LAR* and *ANR*. The key structural gene participating in anthocyanin biosynthesis is different in different plant species. Furthermore, the key structural gene in different tissues within the same plant species also varies. Previous research has shown that the gene involved in the last step, *UFGT*, is the key structural gene involved in anthocyanin biosynthesis and that it determines anthocyanin accumulation in many fruits, such as pear [56], peach [57] and kiwifruit [6]. In a previous study, using transcriptome analysis we concluded that *AaLDOX* is a key structural gene involved in anthocyanin biosynthesis in *A. arguta* fruit [58]. 

Most studies have focused on fruit tissues, whereas flower tissues, have scarcely been documented despite being equally important tissues determining the phenotype of any cultivar. By measurement and comparison of transcription abundance of the 15 structural genes mentioned above in the three different types of *Actinidia* petals under study here, we found that the regulation of *CHS* expression was different from that of other genes. The expression level of *CHS* in *A. eriantha* petals was significantly higher than that in *A. hemsleyana* or in *A. arguta* petals (Figure 4A). Cluster analysis showed that *CHS* was clustered into a single class distinctly separated from the rest of the genes (Figure 4B), possibly indicating that the expression pattern of *CHS* seems to be different from others and *CHS* might be the key gene controlling anthocyanin biosynthesis in *A. eriantha* petals. Therefore, we selected *CHS* as candidate structural gene for subsequent research. Through homologous cloning, we obtained the *AeCHS* complete coding sequence with 1170 bp encoding a protein of 389 amino acids from *A. eriantha* cv ‘Zaoxu’ petal and sequence alignment showed *AeCHS* was highly conserved in *Actinidia* species (Figure 5). Furthermore, subcellular localization experiments conducted by transient injection assays in *N. Benthamiana* leaves showed that AeCHS is located in the cytoplasm, which indicates *AeCHS* is indeed a structural gene encoding chalcone synthase participating in anthocyanin biosynthesis and controlling anthocyanin accumulation. These results prove that the key structural gene responsible for color is different in different plant tissues.

### 4.3. Infiltration Grafting-mediated VIGS

Although the VIGS methodology was reported a long time ago [47], its application is still quite limited in fruit trees compared with other model plant species. There have been few successful reports of VIGS application, such as in apple [50], strawberry [51] and recently in kiwifruit [52]. In perennial woody plants, such as fruit trees, grafting is the main reproductive mode; therefore, here we used the new experimental method known as infiltration grafting-mediated VIGS to silence the target gene in *A. eriantha* petals based on the previous experience with the application of the VIGS method in roses [43]. The petal phenotype of silenced *AeCHS* was obviously white at the upper part of the petal, while the control phenotype, i.e., without silenced *AeCHS*, was the normal red color (Figure 7). Total anthocyanin content of silenced petal *AeCHS* was significantly lower than that of control petals (Figure 8A); furthermore, analysis of the expression level showed that the abundance of *AeCHS* transcripts in the *AeCHS*-silenced petals was less than 80% of that in control petals at twenty days after infiltration grafting (Figure 8B). In addition, the expression level of *LBGs* including *LDOX*, *UFGT* and *UFGGT* involved in anthocyanin biosynthesis in silencing petals was significantly lower than that in control and non-treated petals (Figure 8C). This might suggest silencing of *AeCHS* might reduce anthocyanin biosynthesis and accumulation mainly by repressing expression of *LBGs* in *A. eriantha* petal. However, the specific mechanism needs further study to prove. Since there are different copies for different genes of the anthocyanin pathway, the derived conclusion might be limited by the results of one gene copy. Therefore, it is necessary to understand the expression and function of each gene copy in further research.

**Figure 9:**
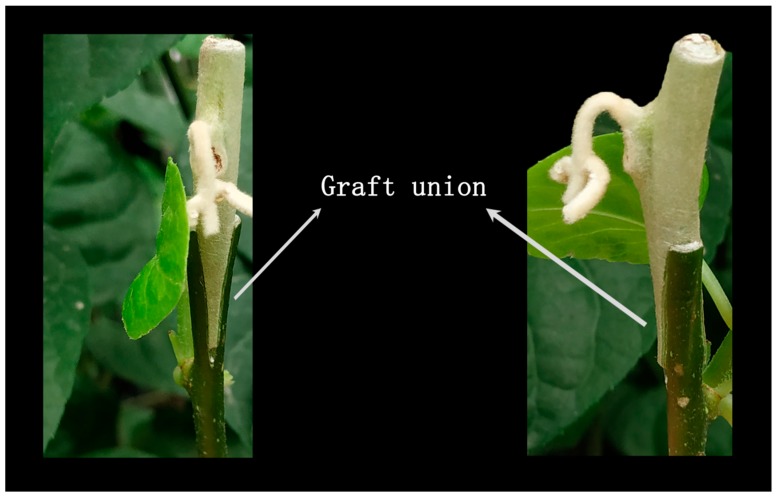
Growth status of scions after removing flowers.

Altogether, these results suggest that infiltration grafting-mediated VIGS might be an effective method to silence a target gene, and silencing *AeCHS* could significantly reduce the number of *AeCHS* transcripts and total anthocyanin content, thus resulting in abnormal coloration of *A. eriantha* petals. After removal of the flowers and the transparent bags from the stalk, the graft union can continue growing healthy (Figure 9). Therefore, we conclude that *AeCHS* is a key structural gene involved in anthocyanin biosynthesis and that it is indispensable for petal normal coloration in *A. eriantha*. However, this conclusion is not consistent with previous studies that confirmed that *UFGT*, and not *AeCHS*, is the key structural gene controlling anthocyanin biosynthesis and accumulation in *Actinidia* red petals [8,9]. The reason for the discrepancy might be the difference in materials used for experimentation, as different materials have different key genes playing a key role in anthocyanin biosynthesis. The specific molecular mechanism leading to this difference needs further study.

## Figures and Tables

**Table 1:** Primers used for RT-qPCR.

Gene	Forward Primer (5′ to 3′)	Reverse Primer (5′ to 3′)
*PAL*	GGACTGGGCTTTTGACAGGA	CAGAGGTGCACCATTCCACT
*C4H*	AGTCCAAATCACAGAGCCCG	GTATCCACCGAGCTTTGCCT
*4CL*	TTGGCCAGGGCTATGGAATG	GCCAGTTTCGGGGTCGATAA
*CHS*	ACAAAGCTCCTTGGTCTCCG	CCCCCTTGTTGTTCTCTGCT
*CHI*	TGCCATTAACGGGCAAGGAA	TTGTAACGGCTTTGGCCTCT
*F3H*	ACCATCACGCTCTTGCTTCA	TGCTTGGTGGTCTGCATTCT
*F3′H*	CACCCTCCTTAACCGTCACC	CGGGCCATGGAAGCTATTGA
*F3′5′H*	GTGGGAAAACCCGCTAGAGT	TCCCATCCTTATGCCTGCAC
*DFR*	CTTCCATGTCGCCACTCGTA	CAGATTGGGGGTGTTGTTGC
*LDOX*	TACCCGGAGGACAAAAGGGA	GAGCCGACCCTCTTCAAGTC
*UFGT*	CGTGGCAATAGCTGAAGCAC	GAGTTCCACCCGCAATGAGT
*UFGGT*	CACGTCCCGGAAACCCTAAA	TGCTCTCCCCAAAATCGCAT
*FLS*	GGCAGTGTACCATCGGTCAA	TGTCATCCCCAACGAGCTTC
*LAR*	GGTTCCTGCCATCGGAGTTT	GAAGTAGGGCCACGAAGCAA
*ANR*	GTACAACGTCCCCACCGATT	TGAAGTAGGCGACGGATTGG
*β-actin*	TGCATGAGCGATCAAGTTTCAAG	TGTCCCATGTCTGGTTGATGACT

**Table 2:** Correlation analysis between content of anthocyanin components precursors and expression level of fifteen genes involved in anthocyanin biosynthesis.

Anthocyanins and Precursors	*PAL*	*C4H*	*4CL*	*CHS*	*CHI*	*F3H*	*F3’H*	*F3’5’H*	*DFR*	*LDOX*	*UFGT*	*UFGGT*	*FLS*	*LAR*	*ANR*
Dihydroquercetin	0.528	−0.738 *	0.432	0.423	−0.746 *	−0.14	0.003	0.135	0.595	0.255	0.208	−0.639	0.14	0.421	−0.793 *
Cyanidin	−0.163	−0.529	0.925 **	0.939 **	−0.625	−0.742 *	0.739 *	−0.387	−0.102	−0.586	−0.585	−0.858 **	−0.665	0.951 **	−0.437
Cyanidin-3-O-galactoside	−0.793 *	0.169	0.657	0.688 *	0.038	−0.904 **	0.984 **	−0.782 *	−0.742 *	−0.947 **	−0.958 **	−0.356	−0.962**	0.720 *	0.253
Cyanidin-3-O-xylose-galactoside	−0.066	−0.633	0.971 *	0.977 **	−0.703 *	−0.672 *	0.584	−0.295	−0.011	-0.528	−0.514	−0.923 **	−0.608	0.965	−0.496
Dihyromyricetin	0.052	−0.720 *	0.960 **	0.960 **	−0.790 *	−0.585	0.558	−0.181	0.099	−0.448	−0.421	−0.961 **	−0.527	0.952 **	−0.591
Delphinidin	−0.055	−0.632	0.953 **	0.966 **	−0.709 *	−0.682 *	0.662	−0.296	−0.016	−0.529	−0.517	−0.921 **	−0.61	0.966 **	−0.497
Delphinidin-3-O-galactoside	−0.122	−0.598	0.985 **	0.995 **	−0.679 *	−0.725 *	0.668 *	−0.349	−0.07	−0.585	−0.573	−0.920 **	−0.664	0.990 **	−0.459

‘*’ indicates correlation is significant at the 0.05 level (2-tailed); ‘**’ indicates correlation is significant at the 0.01 level (2-tailed). The gray shadow mark represents the expression level of candidate gene *CHS* is significantly correlated to 6 anthocyanins at 0.05 and 0.01 level.

## Supplementary Materials

The following are available online at https://www.mdpi.com/2073-4425/10/12/949/s1, Figure S1: Basic information of *AeCHS* used for VIGS.
