# Supplementary material for: Chalcone Synthase-Encoding AeCHS is Involved in Normal Petal Coloration in Actinidia eriantha
Source: Genes (Basel). 2019 Nov 20;10(12):949. doi: 10.3390/genes10120949 (PMC6947247; doi:10.3390/genes10120949)
Supplement: Supplementary file 1 [file genes-10-00949-s001.pdf]

File S1 Basic information of *AeCHS* used for VIGS

>AeCHS-*Actinida eriantha* cv 'Zaoxu'（1170bp；389aa）

ATGGTGACTGTCGAGGAAGTTCGAAGGGCACAGCGGGCCGAGGGACCGGCCACG
GTCATGGCCATCGGAACGGCGACCCCGCCGCACTGCGTTGACCAGAGCACGTATC
CCGATTACTACTTTCGTGTGACCAATAGCGAGCACAAGGCAGAGTTGAAAGAGA
AGTTCCAGCGCATGTGTGACAAATCCATGATCAAGAAGCGTTACATGTACTTGAC
TGAGGAGATCTTGAAAGAAAACCCTAATGTGTGTGCCTACATGGCCCCATCACTT
GATGCTAGGCAGGACATGGTGGTCGTGGAAATTCCCAAATTGGGCAAGGAGGCT
GCCGTCAAAGCCATCAAGGAATGGGGCCAGCCCAAGTCCAAAATTACCCATTTG
GTCTTTTGTACCACTAGCGGGGTCGACATGCCCGGCGCTGACTATCAGCTCACAA
AGCTCCTTGGTCTCCGCCCGTCTGTCAAGCGCCTCATGATGTACCAACAGGGTTG
CTTCGCTGGTGGCACGGTGCTCCGCTTGGCCAAGGACCTAGCAGAGAACAACAA
GGGGGCCCGTGTATTGGTTGTCTGCTCTGAAATCACTGCTGTCACTTTCCGTGGGC
CCAGTGACACCCACCTCGACAGTCTTGTTGGTCAGGCCTTGTTTGGTGATGGTGC
AGCCGCTATTATAGTTGGGGCCGACCCAATTCCCGAGGTTGAGAAGCCCATGTTT
GAGTTGGTTTCGGCGGCCCAAACCATCTTACCGGATAGTGATGGGGCCATCGATG
GACATCTCCGCGAAGTGGGCCTGACCTTCCACCTCCTCAAGGATGTACCTGGGCT
TATTTCCAAAAACATTGAAAAAAGCCTAGTGGAGGCATTCAAGCCCTTGGGCATC
TCGGACTGGAACTCCCTCTTCTGGATCGCACACCCTGGTGGGCCTGCTATTTTGGA
CCAAGTGGAACAAAAATTGGCCCTTAAGCCCGAGAAGCTACGGGCCACGAGGCA
CGTGCTAAGCGAGTACGGTAACATGTCGAGCGCGTGCGTGTTGTTCATTTTGGAT
GAGATGAGGAAGAAGTCTGCCGAAGACGGGCACAAGACCGCCGGTGAGGGGCT
CGAGTGGGGCGTGCTCTTTGGGTTTGGACCCGGGCTTACTGTTGAGACTGTGGTG
CTCCATAGCCTGTGCACTTAG

AeCHS

Specific fragment **307** **389**

> Specific fragment：(83 aa)

CCTGCTATTTTGGACCAAGTGGAACAAAAATTGGCCCTTAAGCCCGAGAAGCTAC
GGGCCACGAGGCACGTGCTAAGCGAGTACGGTAACATGTCGAGCGCGTGCGTGT
TGTTCATTTTGGATGAGATGAGGAAGAAGTCTGCCGAAGACGGGCACAAGACCG
CCGGTGAGGGGCTCGAGTGGGGCGTGCTCTTTGGGTTTGGACCCGGGCTTACTGT
TGAGACTGTGGTGCTCCATAGCCTGTGCACTTAG

Restriction enzymatic site: F-*Eco*R I；R-*Kpn* I

Primers for VIGS:

F- **CCGGAATTCCGG** CCTGCTATTTTGGACCAAGTGG

R- **CGGGGTACCCCG** CTAAGTGCACAGGCTATGGAGC
